# Coronary Whole-heart MR Angiography: Feasibility of Breath-hold Chasing with Diaphragmatic Navigation

**DOI:** 10.2463/mrms.tn.2015-0096

**Published:** 2015-12-22

**Authors:** Hideyuki MATSUTANI, Makoto AMANUMA, Takako SEKINA, Takeshi KONDO, Shigehide KUHARA, Shinichi TAKASE

**Affiliations:** 1 Department of Radiation Technology, Takase Clinic; 2 Department of Radiology, Takase Clinic, 885-2 Minami-Orui, Takasaki, Gunma 370-0036, Japan; 3 Department of Cardiology, Jukokai Central Hospital; 4 Clinical Application Research Center, Toshiba Medical Corporation; 5 Department of Cardiology, Takase Clinic

**Keywords:** coronary magnetic resonance angiography (CMRA), real time motion correction (RMC), coronary artery, SSFP

## Abstract

We propose a simple but novel data acquisition technique for whole-heart coronary magnetic resonance angiography (CMRA). In this technique, the breath-hold chasing MRA, data are collected during breath-hold intervals, with the navigation window manually adjusted to the diaphragmatic level. Compared with the conventional free breathing MRA, this method provided 33% reduction of acquisition time and improved visibility of right coronary artery in 18 normal subjects without any additional software or hardware requirements.

## Introduction

Coronary magnetic resonance angiography (CMRA) has several advantages over computed tomographic angiography (CTA). As it requires no ionizing radiation, it is suitable for assessment of younger patients with coronary artery abnormalities such as congenital anomaly or Kawasaki disease.^1^ As it also needs no contrast media, patients with deteriorated renal function or hypersensitivity to iodine contrast media can be also assessable.^2^ Additionally, patients with severe calcification, for whom evaluation by CTA is often impossible, can be readily assessable.^3^

Today, the so-called whole-heart coronary MRA (WHCMRA) is commonly performed.^4^ While this technique effectively collects three-dimensional (3D) data of all coronary branches under free breathing, one of the major disadvantages is its long acquisition time. We propose a new approach for WHCMRA data collection, breath-hold chasing MRA (BHC-MRA), in which method data acquisition is performed only during the breath-hold interval. Actual acquisition time and the image quality of the method were compared with the conventional free-breathing MRA (FB-MRA).

## Materials and Methods

### Ethics

Written informed consent was obtained from each subject before the examination. This prospective study was approved by our institution’s ethics review board prior to the start of the study.

### Subjects

Between May 1 and July 15, 2015, WHCMRA was performed in successive 18 healthy subjects (16 men, 2 women; 56 ± 10 years old) who understood the purpose and agreed to join this study. Eight to 10 hours before the examination, 25 mg of atenolol was administered orally unless contraindications were present.

### Medical equipment, imaging protocol, and parameters

We used a 1.5T MR unit (Vantage Titan, Toshiba Medical Systems Corporation, Tochigi, Japan) and dedicated 32 channel coil (Atlas SPEEDER coil, Toshiba Medical Systems Corporation, Tochigi, Japan). For image processing and analyzing, 3D work station (Ziostation 2, Ziosoft Inc., Tokyo, Japan) was used.

All localizer and MRA images were obtained using a coherent-type gradient echo sequence (true steady state free precession; True SSFP, Toshiba). Imaging protocol was as follows:

1. 2D orthogonal localizer (2D True SSFP; repetition time [TR]/echo time [TE], 3.5/1.7; flip angle [FA], 50°)
2. Axial mutislice 2D field echo for 3D localizer (2D True SSFP; TR/TE, 3.4 ms/1.7 ms; FA, 50°)
3. Cine 4-chamber images (2D True SSFP; TR/TE, 3.4 ms/1.7 ms; FA, 69°)
4. FB-MRA (3D True SSFP; TR/TE, 4.5 ms/2.25 ms; FA, 115°)
5. BHC-MRA (3D True SSFP; TR/TE, 3.4 ms/2.25 ms; FA, 115°)

The basic imaging parameters of BHC-MRA and FB-MRA were identical. We used 3D True SSFP with a frequency-selective fat suppression technique (FatSat Standard) and T_2_ preparation pulses. Parallel imaging (SPEEDER, Toshiba) factor was 2.0. Field of view (FOV) was 330 × 190–220 mm with in-plane resolution of 1.5 × 1.5 mm. Partition thickness was also 1.5 mm. Using a high resolution reconstruction protocol, data were interpolated to 0.75 mm isovoxels. Segment number of data acquisition varied from 1 to 3, determined by the static interval of the coronary arteries observed on cine four-chamber images. Date acquisition interval was principally at mid-diastolic phase. When heart rate was high, data were collected at end-systolic phase.

For respiratory gating, diaphragmatic navigation (real time motion correction: RMC^5^) was used with a navigation window of 3.28 mm. All studies were performed with supplemental 3 L/min oxygen administration via a nasal tube.

### FB-MRA

Patients were instructed to be comfortable to make their breaths as stable as possible. At the beginning, we observed the respiratory pattern of the subject on RMC monitor and set an optimum navigation window level at expiration. Basically the level of the window was not changed during the scanning. However, when the diaphragmatic level moved due to fluctuations of respiration pattern and the ratio of effective sampling became lower than 10%, we manually changed the position and range of navigation window so that we could obtain the effective signals more frequently.

### BHC-MRA

**Fig. 1.**
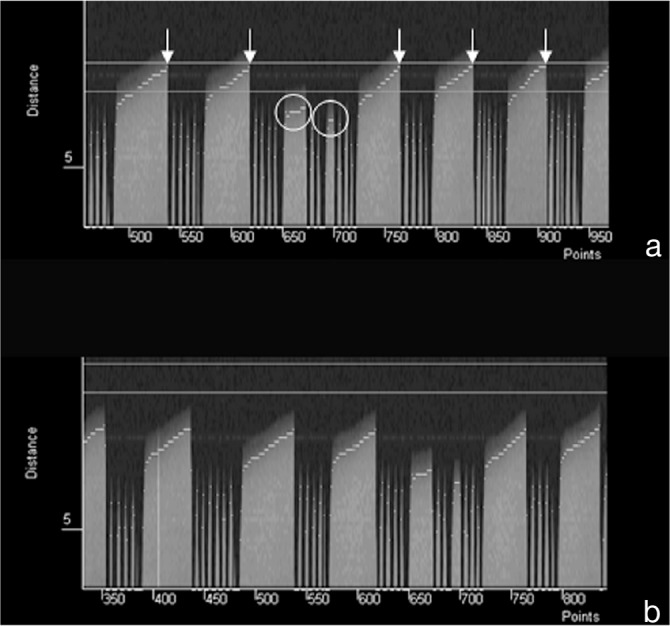
Data collection of breath-hold chasing magnetic resonance angiography (BHC-MRA). (**a**) Navigation window during breath-holding (BH). When the diaphragmatic level shifted outside of the navigation window, the BH was released (arrows). When the diaphragmatic level was unusually shifted from the ordinary levels (circles), the BH was immediately released, too. (**b**) Navigation window during breath relaxing. After releasing BH, sampling window was shifted far above the diaphragm to avoid unfavorable data mixing.

Breath-hold instruction was given to the subject through a headset by the operator observing the RMC monitor. In advance, subjects were instructed to hold his/her breath at expiration as long as possible and release when they felt to keep breath-holding difficult. In the instruction, importance of constantness was stressed rather than length of breath-holding. With each breath-holding, navigation window level was set manually by the operator. When the diaphragmatic level was unusually shifted from the ordinary levels, or it shifted outside of the navigation window, the breathe-holding was immediately released (Fig 1a). While the fluctuation of diaphragmatic breath-hold level is usually within the range of 10 mm, the allowable range of variation of navigation window level was more than 5 cm. After releasing, to avoid unfavorable data mixing, sampling window was shifted far above the diaphragm (Fig. 1b). After releasing breath-hold, relaxed respiration was maintained for several 10 seconds to rejuvenate the patient from increased heart rate. The same procedures were repeated until all data were collected.

### Imaging time measurement

Actual data collection time of each method was recorded. At the same time, the sampled navigation echo data were recorded.

### Subjective analysis

Assessment of image quality was independently made by one radiologist and one radiographer, who have experienced more than 200 coronary MRA readings, thus the final decision was made by consensus of the two. Curved planar reconstruction (CPR) images of the main three coronary branches (#1–3, #5–8, #11 & 13 according to the American Heart Association classification^6^) were reconstructed, and visibility and sharpness of them were assessed using a three-point scale as below.

Excellent: excellent definition without blurring
Acceptable: well defined with mild blurring without non-assessable segments
Unacceptable: poorly defined with substantial blurring, containing at least one segment which could not be confidently evaluated.

Images were randomly reordered by a coordinator (T.S.) and the readers are blinded to the subject’s name and type of data collection of the images. The same reading was again performed 2 weeks later with an altered reading order by the coordinator. Based on these judgments, intraobserver variability and interobserver variability were calculated.

### Quantitative analysis of image contrast

On both FB-MRA and BHC-MRA images, signal intensity and contrast of the arterial blood were assessed. Arterial signal was measured in ascending aorta (SIa) and descending aorta (SId) to avoid a partial volume effect due to a small size of the coronary arterial lumen. Signal intensity of spinae muscles (SIm) was also measured as a reference of contrast in each case. To ensure accurate signal comparison, regions of interest (ROIs) for signal measurement placed on FB-MRA images were copied and pasted on the corresponding BHC-MRA images. The contrast ratios of aortic lumen and muscle were calculated as (SIa-SIm)/SIm and (SId-SIm)/SIm.

### Statistical analysis

Metric data were expressed as mean ± standard deviation (SD). For comparison of numerical values between two independent groups, the Student’s *t*-test was used. For categorical data analysis, Wilcoxon sign rank test was used. Differences were considered to be statistically significant at *P* < 0.05. With regard to the reliability (reproducibility) of intraobserver variability and interobserver variability, the κ value was calculated. A commercially available software package (SPSS version 22, IBM, Armonk, NY, USA) was used to perform statistical analysis.

## Results

All subjects took 25 mg of atenolol before the examination and the heart rate was 59.6 ± 7.6 bpm. The data collection phase of MRA was mid-diastole in 17 subjects and end-systole in 1 patient. Seventeen of 18 subjects underwent both FB-MRA and BHC-MRA successfully. One patient failed to perform FB-MRA due to multiple premature atrial contractions (PACs) but he could undergo BHC-MRA successfully.

Following comparisons were made in the 17 patients excluding the above case.

### Imaging time

**Fig. 2.**
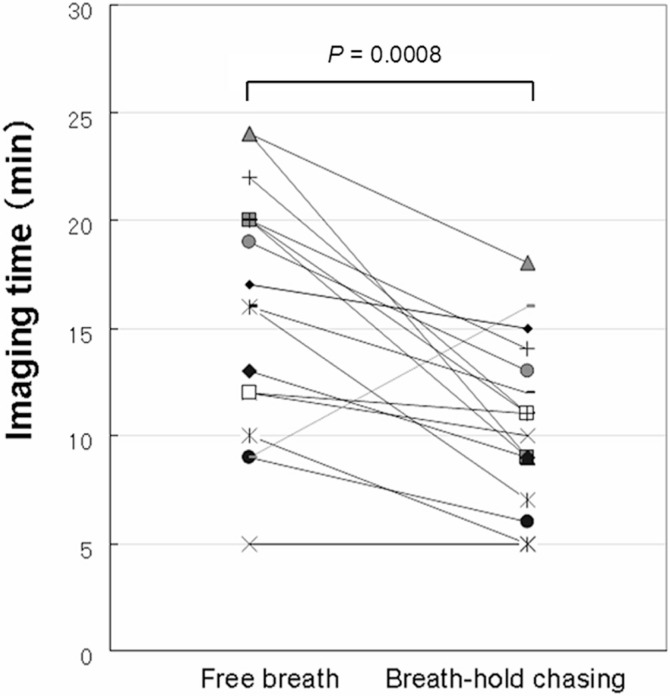
Imaging time for whole-heart coronary magnetic resonance angiography (WHCMRA) in 17 subjects: compared with the acquisition time of free breathing MRA (FB-MRA), that of breath-hold chasing MRA (BHC-MRA) was shorter in 15 cases, unchanged in 1 case, and longer in 1 case. Overall, BHC-MRA showed significantly shorter acquisition time for WHCMRA (*P* = 0.0008).

**Fig. 3.**
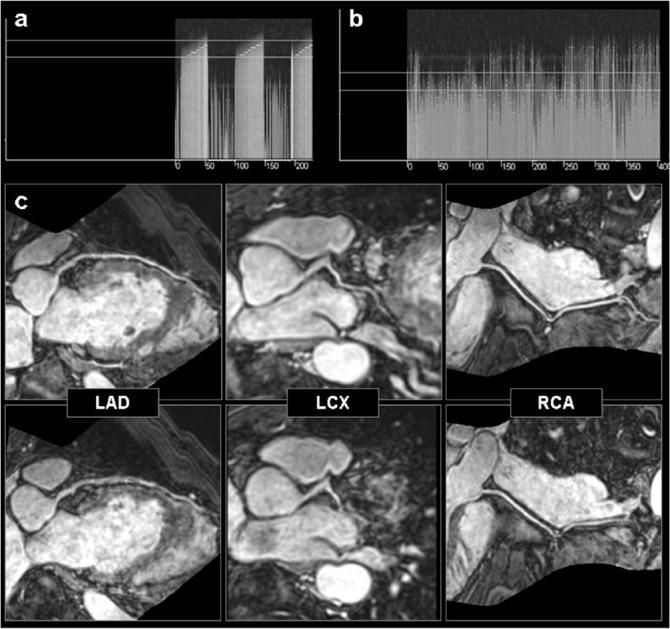
Breath-hold chasing magnetic resonance angiography (BHC-MRA) and free breathing MRA (FB-MRA) in 50-year-old male. (**a**) Navigation echo monitor image of BHC-MRA. Note all data were collected within 3 breath-hold intervals (5 minutes). (**b**) Navigation echo monitor image of FB-MRA. Date acquisition required 10 minutes. (**c**) Curved planar reconstruction images of BHC-MRA (upper level) and FB-MRA (lower level). Image quality of all three branches were judged as “excellent” on BHC-MRA, and judged as “acceptable” on FB-MRA. (As undetectable distal left circumflex branch was considered due to hypoplasty on FB-MRA, its visibility was judged as “acceptable”.) Note, excellent motion-free image of right coronary artery (RCA) on BH-MRA.

Actual imaging time to collect all 3D data was 15.8 ± 5.9 minutes in FB-MRA and 10.6 ± 3.9 minutes in BHC-MRA. BHC-MRA showed a shorter acquisition time with 33% reduction (*P* = 0.0008, Fig. 2). Figure 3 demonstrates a representative case of navigation echo recording of both methods. On FB-MRA, navigation echo distributed randomly and sampling rate of available echo with 3.28 mm-navigation window was between 20% and 40%. On the other hand, BH-CMRA showed significantly effective data sampling during breath-hold and the overall sampling rate was between 40% and 60%, leading to the shorter acquisition time.

### Subjective analysis of image quality

**Fig. 4.**
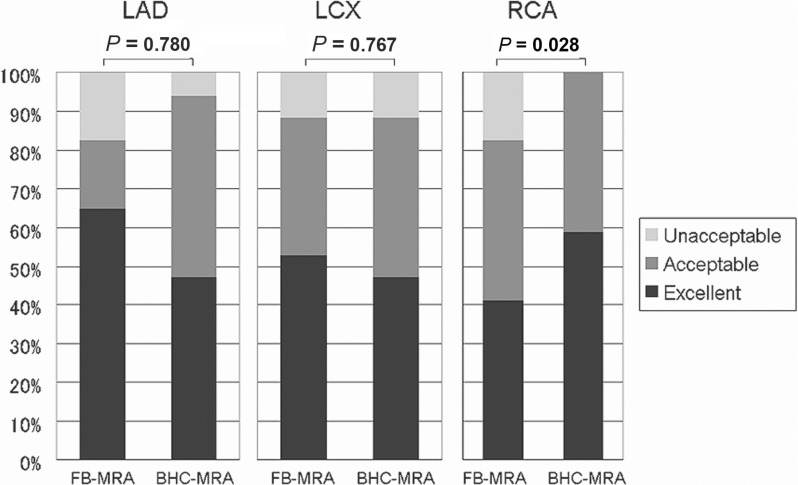
Subjective analysis of visibility and sharpness of coronary branches. Breath-hold chasing magnetic resonance angiography (BHC-MRA) provided superior image quality of right coronary artery (RCA). FB-MRA, free breathing MRA; LAD, left anterior descending artery; LCX, left circumflex artery

Figure 4 represents results of subjective analysis of image quality. As for visibility and sharpness of left anterior descending branch (#6–8) and circumflex branch (#11 & 13), FB-MRA and BHC-MRA showed no statistically significant difference (*P* = 0.780 and 0.767, respectively). On the other hand BHC-MRA showed a significant superior image quality of the right coronary artery (#1–3, *P* = 0.028, Figs. 3, 4). Overall, there was no significant difference between the two methods (*P* = 0.553).

Statistical analysis to evaluate reproducibility in determination of image quality showed that intraobserver variability was κ = 0.794 and intraobserver variability was 0.710.

### Contrast ratio

In 17 subjects, contrast ratio between the ascending aorta and muscle was 2.8 ± 0.7 on FB-MRA and 2.9 ± 0.7 on BHC-MRA. The ratio between the descending aorta and muscle was 2.8 ± 0.7 on FB-MRA and 2.9 ± 0.7 on BHC-MRA. There were no statistically significant in either of them (*P* = 0.712 and *P* = 0.847, respectively).

## Discussion

In WHCMRA, 3D volume data are obtained using the diaphragmatic navigation under free breathing.^4,7^ The data acquisition time largely depends on the respiratory condition. When the respiration is unstable, number of usable echo decreased, resulting in prolongation of the examination time and therefore, deterioration of image quality.

With several technical developments such as parallel imaging, 3T magnet, and 32 channel multi-coil, examination time of WHCMRA have been reduced.^8^ Ultimately even one-breath-hold scanning has recently been reported combining these advanced technologies.^9^ However, these hardware and software are not always available and the one-breath-hold imaging is obtained at the expense of spatial resolution. Explanation and practice of stable respiration are important but not always effective. Usefulness of tight abdominal belt is reported^10^ but it sometimes make patients feel uncomfortable and is not always effective.

To improve the effectiveness of data collection of WHMRA we have examined a simple, but novel approach. On this method, we named breath-hold chasing MRA, navigation window was manually moved to adjust the patient diaphragmatic level during each breath-holding. As the diaphragmatic level was almost always within the navigation window during the period, this method provided effective data collection even if almost no data were collected during the relaxing phase, leading to the significant acquisition time reduction (*P* = 0.0008). One case showed a prolonged examination time (Fig. 2). This is due to an inconsistent breath-hold and a relatively stable free breathing of the subject.

As for image quality, right coronary artery showed statistically significant improvement of the visibility and sharpness while no improvement was noted in left coronary branches. As the number of subjects was limited, we are not sure which factor was responsible for the improved image quality of right coronary artery. It could be due to reduced blurring secondary to shorter acquisition time, improved consistency of diaphragmatic level due to breath-hold, or other reason. Moreover, why improved visibility was observed in only the right coronary artery is also unclear.

As the subjects were normal healthy volunteers, their breathing pattern at relax phase were generally static, so the degree of improvement could have been underestimated. Contrary, as they could hold their breath longer than patients, there was also a possibility that the improvement could be overestimated. Therefore, the actual feasibility of this technique should be evaluated in clinical cases.

One subject was able to be imaged only by BHC-MRA. While the data collection was canceled on the way of scanning as the calculated remaining time exceeded 60 minutes on FB-MRA, data collection successfully finished in 11 minutes on BHC-MRA. If BHC-MRA can improve the technical success rate of WHCMRA, it will be an important advantage and should be also clarified in clinical cases.

As decision of the timing and level of manual navigation window setting were operator-dependent, this method requires some experiences. However, as the procedure itself is simple, the skills would be easily mastered after trials of several cases.

In this study, oral β-blocker was used in all cases as the prolongation of slow filling phase is important for data collection in BHC-MRA. In case of onsite intravenous administration, ultra-short acting β-blocker such as landiolol is useful for CTA but of limited value for WHCMRA (Ono Pharmaceutical Co., Osaka, Japan) as it is only effective for several minutes. The use of longer-acting agents such as atenolol or bisoprolol is desirable, which could be considered as one of the limitations of BHC-MRA. However, except for patients with bronchial asthma or heart failure, β-blocker is an extremely safe premedication^11^ and even long-acting agents can be used without specific discretion.

## Conclusion

Feasibility of BHC-MRA was evaluated in 18 normal subjects. Compared with the conventional FB-MRA acquisition time has decreased by 33% and visibility and sharpness of right coronary artery were improved. Without any specific hardware or software refinement, BHC-MRA can reduce imaging time and improve image quality of WHCMRA.
